# Towards Integrated Surveillance of Marine Brucellosis: Diagnostic and Phylogenetic Assessment of *Brucella ceti* in Stranded Dolphins of the Western Mediterranean Sea

**DOI:** 10.1155/tbed/2075116

**Published:** 2026-01-31

**Authors:** Ignacio Vargas-Castro, Sara Andrés-Barranco, José Luis Crespo-Picazo, Laura Torre-Fuentes, Mª Ángeles Jiménez-Martínez, Marta Hernández, Manuel Arbelo, Julio Álvarez, Pilar María Muñoz, Vicente Marco-Cabedo, María Jesús de Miguel, Débora López, Marta Muñoz-Baquero, Daniel García-Párraga, José Ángel Barasona

**Affiliations:** ^1^ Animal Health Department, Complutense University of Madrid, Madrid, Spain, ucm.es; ^2^ VISAVET Health Surveillance Centre, Complutense University of Madrid, Madrid, Spain, ucm.es; ^3^ Departamento de Ciencia Animal, Centro de Investigación y Tecnología Agroalimentaria de Aragón (CITA) - Instituto Agroalimentario de Aragón-IA2 (CITA-Universidad de Zaragoza), Zaragoza, Spain; ^4^ Research Department, Fundación Oceanogràfic de la Comunitat Valenciana, Valencia, Spain; ^5^ Department of Animal Medicine and Surgery, Complutense University of Madrid, Madrid, Spain, ucm.es; ^6^ Department of Pathological Anatomy, Microbiology, Preventive Medicine and Public Health, Legal and Forensic Medicine, Faculty of Medicine, University of Valladolid, Valladolid, Spain, uva.es; ^7^ Veterinary Histology and Pathology, Atlantic Center for Cetacean Research, University Institute of Animal Health and Food Safety (IUSA), University of Las Palmas de Gran Canaria, Canary Islands, Spain, ulpgc.es

**Keywords:** blocking ELISA, *Brucella ceti*, cetaceans, diagnosis, sequence type, surveillance, WGS

## Abstract

Reports of brucellosis in free‐ranging cetaceans are increasing worldwide, particularly in the Mediterranean Sea. To enhance diagnostic accuracy and epidemiological understanding of cetacean brucellosis in the Western Mediterranean Sea, we analyzed bacteriological, serological, and molecular data from 30 cetaceans belonging to three different species stranded along the coast of the Valencian Community (Spain) between 2011 and 2021. *Brucella ceti* infection was confirmed by bacteriological isolation in 14 animals (46.7%) and by genus‐specific qPCR in 15 cases (50%), with some discrepancies between methods. When feasible, serological analyses were performed using a commercial blocking ELISA (bELISA) and/or the Rose Bengal agglutination test (RBT). In the absence of ELISA tests properly validated for its use in marine mammals, we assessed the optimum dilution and cut‐off of this ELISA kit using panels of gold‐standard sera from culture‐positive and brucellosis‐free dolphins. From a pathological perspective, 12 infected animals showed moderate to severe meningoencephalitis or meningoencephalomyelitis with lymphoplasmacytic infiltration. Additionally, whole‐genome sequencing (WGS) enabled the identification of two sequence types (STs), ST26 and ST49, indicating phylogenetic divergence. Our findings provide new insights into the phylogenetics of *B. ceti* and highlight the particular susceptibility of striped dolphins to this bacterium. The study also evidences the need for proper validation of the indirect diagnostic methods used for surveillance and seroepidemiological studies of brucellosis in marine mammals.

## 1. Introduction

*Brucella* spp. bacteria are Gram‐negative, intracellular pathogens, predominantly zoonotic, with the ability to infect a wide range of hosts [1]. Since the first report of *Brucella* isolations from marine mammals (seals, porpoises, and dolphins) in 1994 [2, 3], this pathogen has been detected in a wide variety of marine mammal species [4]. In 2007, two species of *Brucella* identified in marine mammals were named *Brucella ceti* and *Brucella pinnipedialis*, reflecting their predominant association with cetacean and pinniped hosts, respectively [5]. Subsequent molecular analysis further classified these species into five distinct sequence types (STs): ST23, ST24, ST25, ST26, and ST27. Isolates of *B. ceti* have been associated with ST23, ST26, and ST27, while ST24 and ST25 have been linked to *B. pinnipedialis* [6–10]. However, some genetic studies suggest that ST27 may not fit neatly within the current classification of either *B. ceti* or *B. pinnipedialis* [8, 11].

The most commonly reported lesion associated with brucellosis in cetaceans is meningitis or meningoencephalitis [2, 11–23]. However, the infection has also been linked to a range of other pathological conditions, including reproductive pathologies, such as abortion, stillbirth, placentitis, placental abscesses, orchitis, and epididymitis [2, 11, 17–19, 24–28]; bone abnormalities, such as osteoarthritis, discospondylitis, and vertebral osteomyelitis [11, 17, 24, 29, 30]; respiratory lesions as pneumonia and lung abscesses [20–22, 24, 31, 32]; hepatomegaly, splenomegaly, and lymphadenomegaly with associated necrosis and inflammation [20, 24]; and other manifestations as subcutaneous abscesses [17, 33]. However, pathological changes associated with brucellosis may not always be evident in infected individuals [4, 6]. Different reasons have been suggested to explain the absence of clinical signs, including (i) the ability of the animal to control the infection and become an asymptomatic carrier [6], (ii) variability in the pathogenicity among *B. ceti* strains [8], and/or (iii) differences in susceptibility among individual animal or species [15].

Brucellosis in terrestrial animals as domestic ruminants and pigs is considered occupational zoonosis and, in developed countries, is classified as notifiable disease under surveillance, control, or eradication programs [34]. Although human infections with marine brucellae species have been reported [35–37], marine brucellosis is a neglected zoonosis [6]. Nevertheless, the increasing frequency of interactions between cetaceans and humans [18, 38–42] highlights the need for continued surveillance and monitoring studies to better understand cetacean brucellosis from a One Health perspective [20, 23, 42–45].

The diagnosis of marine brucellosis relies on the observation of suspicious pathological lesions in stranded specimens and, whenever possible, the subsequent bacteriological or molecular analysis of selected tissues. Serological analysis might be also useful to detect suspicious animals in a routine diagnosis. However, to our knowledge, no serological tests have been validated for the specific diagnosis of brucellosis in marine mammals; thus, commercial tests designed for terrestrial animals are often applied to marine mammals [22, 44, 46, 47] without prior validation, which raises concerns about their diagnostic reliability.

On the other hand, previous studies have demonstrated the utility of whole‐genome sequencing (WGS) of marine *Brucella* (*B. ceti* or *B. pinnipedialis*) to enhance our understanding of these pathogens [20, 22, 43, 48–50]. Nevertheless, further information on the genetic variability of field strains of marine *Brucella* are needed to establish robust genetic markers for identifying epidemiologically related isolates [22].

In order to improve our understanding of diagnostic methods and the occurrence of cetacean brucellosis in the Western Mediterranean Sea, we present data from 30 cetaceans, belonging to three different species, stranded along the coast of the Valencian Community (Spain) between 2011 and 2021, in which *Brucella* infection was suspected either based on clinical signs or on lesions compatible with the disease. This investigation involves different diagnostic methods, including direct qPCR, bacterial culture, serological tests, and histopathologically examination. Moreover, *B. ceti* isolates obtained from culture‐positive cases were subjected to WGS and comprehensive phylogenetic analyses (Supporting Information 1: Table S1). This allowed us to study the genetic heterogeneity among isolates and provided insights into the genomic diversity of field strains of *B. ceti*.

## 2. Materials and Methods

### 2.1. Stranded Dolphins Investigated

A total of 30 cetaceans stranded between 2011 and 2021 in which *Brucella* infection was suspected were investigated. The animals belonged to three species: striped dolphin (*Stenella coeruleoalba*) (*n* = 25), Risso’s dolphin (*Grampus griseus*) (*n* = 3), and common bottlenose dolphin (*Tursiops truncatus*) (*n* = 2).

Necropsies were conducted in accordance with the standard procedures of the European Cetacean Society [51], and the sex and age categories were determined. The classification of these individuals into age groups (adults, juveniles, calves, and neonates) was performed following established criteria in the literature [52–58].

A suspected diagnosis of brucellosis was considered when clinical signs such as uncoordinated swimming, circling, lateralization, buoyancy disturbances, reduced responsiveness to stimuli, lethargy, poor body condition, closed eyes, opisthotonos, tremors, and/or tonic‐clonic seizures were detected in live‐stranded animals or when lesions compatible with the disease were observed during pathological examination (Section 2.2). Subsequently, tissue, fluid, and, whenever possible, serum samples were submitted to bacteriological, molecular, and serological analyses (Supporting Information 2: Table S2).

### 2.2. Histopathological Analysis

Tissues included cerebrum, cerebellum, brain stem, spinal cord, oral mucosa, tonsils, trachea, lung, heart, liver, stomach (glandular and nonglandular), pancreas, intestine, kidneys, spleen, gonads, lymph nodes, skin, and genital mucosa. These tissues were collected routinely during necropsy for each of the animals included in the histopathological study. Samples were fixed in 10% neutral buffered formalin, embedded in paraffin, sectioned at 4 ± 2 µm, and stained with hematoxylin and eosin (H&E) following standard laboratory procedures and examined through a light microscope by a trained pathologist. A total of 23 of the 30 cetaceans were included for histopathological analysis.

### 2.3. Bacteriological Examination and Identification of *Brucella* Isolates

Tissue samples and fluids were processed following the protocol described elsewhere [59]. Briefly, tissue samples (Supporting Information 2: Table S2) were degreased, superficially sterilized by gentle burning, and homogenized in minimal amount of buffer using a Stomacher. At least 0.5 mL of each homogenate or fluid (cerebrospinal fluid (CSF) or urine) was cultured by duplicate on CITA and Farrell selective media plates and incubated during 5–7 days at 37 °C in a 10% CO_2_ atmosphere. Suspicious colonies were identified as *Brucella* using standard procedures [60] and the Bruce‐ladder multiplex PCR [61], which allows to identify the main *Brucella* species, including those affecting marine mammals. Bacterial DNA was extracted using the Speedtools Tissue DNA Extraction kit (Biotools, Madrid, Spain).

To distinguish between *B. ceti* and *B. pinnipedialis*, we used a multiplex PCR adapted from López‐Goñi et al. [62], with the following two pairs of primers: TCA ACT GCG TGA ACA ATG CT (f)/GCG GGC TCT ATC TCA AGG TC (r) and CGT CAA CTC GCT GGC CAA GAG (f)/GCA GGA GAA CCG CAA CCT AA (r). All the isolates were also typed by PCR‐RFLP of the Omp2b locus [63].

### 2.4. Molecular Diagnosis by Direct qPCR

Direct molecular diagnosis was performed by qPCR on the following tissue samples, whenever possible: CSF, cerebrum, cerebellum, spinal cord, liver, spleen, pharyngeal tonsils, lung, mediastinal lymph node, testis, uterus, umbilical cord, amniotic fluid, mammary gland, and milk (Supporting Information 2: Table S2). For this, all samples were homogenized using stainless steel 4.8‐mm beads (Next Advance, New York, USA) after being added to phosphate‐buffered saline (PBS) at a 1:10 proportion. DNA was extracted from the homogenates using the High Pure Template Preparation Kit (Roche Diagnostics, Mannheim, Germany), according to the manufacturer’s instructions, and subsequently submitted to the real‐time PCR targeting the IS711 insertion sequence as previously described [64]. Ultrapure water was used as a negative control, while DNA from the *Brucella melitensis* B115 vaccine strain was used as a positive control.

The PCR products from positive tissues were purified using the QIAquick PCR Purification Kit (Qiagen, Hilden, Germany), and amplicons were completely sequenced by Sanger DNA sequencing (Macrogen Inc., Madrid, Spain). The amplicon identities were confirmed with BLAST (https://blast.ncbi.nlm.nih.gov/Blast.cgi).

Positive and negative predictive values (PPV and NPV) of qPCR were calculated on a per‐animal basis, using bacterial culture as the reference standard (a dolphin was considered positive when at least one tissue sample tested positive by the corresponding method).

### 2.5. Serology

Whenever possible (i.e, when enough amount of nonhemolyzed serum was available), sera were analyzed by the Rose Bengal agglutination tests (RBT) (following the standard procedure used for ruminants [65].

Available sera were also tested using the commercial kit INgezim Brucella Compac 2.0 (Gold Standard Diagnostics, Madrid, Spain), a multispecies blocking ELISA (bELISA, often misclassified as competitive ELISA) designed to detect specific antibodies to LPS of *Brucella* spp. in bovine, ovine, caprine, and porcine sera. In order to assess the performance of this bELISA for its use in dolphins, we tested sera from eight *B. ceti* culture positive dolphins as a panel of positive gold‐standard sera (Sc 25.03.11, Sc 18.06.11, Sc 07.07.11, Sc 29.07.11, Sc 25.10.13, Sc 15.08.14, and two additional striped dolphins stranded in 2022 but not included in the genetic study) and 84 brucellosis‐free dolphins. These brucellosis‐free animals were reared under human care in four different zoologic centers from Spain and Portugal (Zoo de Madrid *n* = 32], Zoomarine Algarve [*n* = 26], Oceanogràfic [*n* = 19], and Mundomar [*n* = 8]) under controlled environment, with strict biosecurity protocols and no contact with wild specimens, a complete absence of clinical symptoms compatible with brucellosis in their medical history and negative results in previous serodiagnostic tests (RBT).

The optimum serum dilution and cut‐off were determined to achieve total specificity (i.e., absence of false positive results in the brucellosis‐free population) and the maximum associated sensitivity to detect culture‐positive animals. For this, different serum dilutions (1/5, 1/10, 1/20, and 1/40) were analyzed by duplicate and following the manufacturer’s instructions. Optical densities (OD_450 nm_) were automatically assessed (Labsystems Multiskan RC), and the inhibition percentages (PI) were calculated, using the formula: PI = 100 × [1 − (OD_450 nm_ sample/OD_450 nm_ negative control)].

### 2.6. Whole‐Genome Sequencing and Bioinformatics

For the WGS analysis, we analyzed 14 isolates, each obtained from a different animal. Additionally, in three of these dolphins, a subset of isolates obtained from two distinct tissues within the same animal were analyzed to uncover potential single nucleotide polymorphism (SNP) variations occurring within the same animal.

Bacterial DNA was purified from 17 axenic cultures using the Qiagen DNA Blood and Tissue Kit, following the manufacturer’s instructions for “Gram‐negative bacteria” protocol (Qiagen, Hilden, Germany). DNA concentration was quantified using a Qubit fluorometer (Invitrogen), which was also employed for subsequent library quality assessments. WGS libraries were prepared from 1 ng of bacterial DNA using the Nextera XT DNA Library Preparation Kit (Illumina, USA). Each library was normalized to a final concentration of 4 nM to obtain equimolar pooling prior to sequencing on a MiSeq device (Illumina).

Raw sequencing reads were filtered out using Trimmomatic [66] to remove adapter sequences and low‐quality reads. Quality‐filtered reads were then assessed using FastQC prior to genome assemblies generated using SPAdes, and their quality was evaluated with QUAST [67]. To determine the STs of the assembled genomes, multilocus sequence typing (MLST) was performed using the MLST software developed by T. Seemann (https://github.com/tseemann/mlst), in conjunction with the publicly available PubMLST Brucella database (https://pubmlst.org/brucella/).

### 2.7. Phylogenetic Analysis

For the phylogenetic analysis, we incorporated sequence data from four *B. pinnipedialis* and 19 *B. ceti* strains included in previous studies [20, 43, 68]. This inclusion allowed the evaluation of the genetic relationships between the strains described in our study and those available from different parts of the world, including the United States, Costa Rica, Norway, Scotland, Italy, Portugal, and Spain. Additionally, *B. abortus* (GenBank Accession Numbers NC_006932.1 and NC_006933.1) was included in the analysis as an outgroup to root the tree (Supporting Information 3: Table S3). For public genome assemblies, paired‐end reads were simulated from genome assemblies with ART (MountRainier) [69]. All reads were mapped against the reference genome of *B. ceti* (GenBank Accession Number NC_022905.1 and NC_022906.1) using BWA [70] with default parameters. SAMtools [71] was used for sorting and compressing the obtained SAM files into BAM files. The variant calling was performed applying “mpileup” and “call” options with BCFtools [72]. The resulting SNPs were filtered by removing those with a base quality < 30 and a mapping quality < 30. Consensus sequences were then created from the corresponding variant call format (VCF) file using BCFtools for each strain. Concatenated consensus sequences were used to generate a maximum likelihood phylogenetic tree using RAxML [73]. The tree was constructed using the general time‐reversible substitution evolutionary model with gamma correction and 1000 bootstrap replicates. The tree was rooted using the sequence from *B. abortus* and visualized using iTOL [74].

The number of high‐quality SNPs (hqSNPs) between strains from different tissues was obtained from the VCF files and verified by manual review of the alignments using the Integrative Genomics Viewer (IGV) tool [75] to accurately assess their differences.

## 3. Results

### 3.1. Diagnostics

Diagnostic results obtained by bacterial culture, qPCR, and serological tests (RBT and bELISA) are compiled in Table 1 (detailed individual results) and Supporting Information 2: Table S2 (cross‐tabulation of diagnostic results).

**Table 1:** Cross‐classified diagnostic results for brucellosis in 30 dolphins.

Number of animals	(%)	Culture	qPCR	RBT	bELISA
4	(13.3)	1	1	1	1
7	(23.3)	1	1	NA	NA
1	(3.3)	1	0	NA	NA
4	(13.3)	0	1	NA	NA
2	(6.7)	1	0	0	1
11	(36.7)	0	0	NA	NA
1	(3.3)	0	0	0	0

*Note:* NA: serum not available.

*B. ceti* infection was confirmed by bacterial culture and subsequent PCR (Bruce‐ladder and marine multiplex PCR) in 46.7% (14/30) of the dolphins examined. Out of the 155 samples cultured, the bacterium was isolated in 25 (16.1%). The qPCR, using a cycle threshold (Ct) of 40 as the cut‐off, followed by sequencing, yielded positive for *Brucella* spp. in 50% (15/30) of the animals, with some discrepancies with respect to bacterial culture: 11 dolphins were positive by both methods, three only by culture, and four only by qPCR, resulting in a concordance (Po) of 77%. Among the 238 tissue samples tested by qPCR, 36 (15.1%) were positive, with Ct values ranging from 31.28 to 39.

Using bacteriology as the gold‐standard, qPCR showed a PPV of 73.3% (IC95%: 44.9%−92.2%) and a NPV of 80% (51.9%−95.7%) when calculated per animal.

Considering the proportion of positive samples by organ or sample analyzed, the bacteria was more frequently isolated from the CNS (14/39, 35.9%), followed by spleen (6/23, 26.1%), tracheobronchial lymph node (2/23, 14.8%), and pharyngeal tonsils (1/14, 7.14%). The proportions of *Brucella*‐positive samples by qPCR, from highest to lowest, were CNS (23/83, 27.7%), reproductive tissues (3/19, 15.8%, including amniotic fluid, uterus, mammary gland, and fetal samples), pharyngeal tonsils (3/23 = 13.0%), mediastinal lymph node (2/23 = 8.70%), spleen (2/26, 7.69%), liver (2/29, 6.90%), and lung (1/27, 3.70%).

Table 2 summarizes the detailed findings of the neuropathological analysis. Inflammation in the CNS was observed in 18 of the 23 animals available for histopathological examination; 12 of which corresponded to dolphins that tested positive for brucellosis by either culture or qPCR. Lesions in CNS in these 12 animals were consistent and involved moderate to severe meningoencephalitis, with extensive inflammatory infiltrates that expanded the leptomeninges four to 12 times the normal width (Figure 1a–f). Inflammatory cells consisted of lymphocytes, plasma cells, moderate numbers of histiocytes, and lesser neutrophils (Figure 1e–i). The areas that were most consistently affected were cerebrum, brain stem, and spinal cord, and in lesser measure the cerebellum. Overt perivascular cuffing was frequent, with similar inflammatory cells and occasional gemistocytes (Figure 1g,h). Other on and off findings were gliosis, with reactive astrocytosis and microgliosis, neuronal satelitosis, and neuronal necrosis, mainly in the deep cerebral cortex. Meningomyelitis and meningopolyradiculoneuritis were consistently observed in *B. ceti*–positive cases when nerve roots from the spinal cord were available. Occasional ventricular inflammation was also observed. Lesions were similar regardless of the strain of *B. ceti* (ST26 or ST49). Only one positive case (unconfirmed by culture) exhibited mild cerebral gliosis as the sole CNS lesion, while one case had severe meningoencephalitis resembling that of *Brucella*‐positive cases despite resulting negative for brucellosis assays. No lesions associated with brucellosis were observed in other organs or tissues.

Figure 1Histopathology of *Brucella* sp. infection in leptomeninges, brain, spinal cord, and nerve roots. Hematoxilin‐eosin stain. (a, b) Power field (PF) x4: severe, diffuse leptomeningeal inflammatory infiltrates ( ^∗^) expanding over 10 times the normal width of the meninges and infiltrating the subjacent neuropil and adjacent cranial nerve roots (arrow). (c) PF x4: moderate diffuse, cerebellar leptomeningeal infiltrates of numerous lymphocytes, plasma cells, macrophages, and sparse neutrophils. (d) PF x4 and (e) PF x10: severe, diffuse, lymphoplasmacytic, and histiocytic meningomyelitis ( ^∗^) and polyradiculoneuritis consisting of similar inflammatory infiltrates surrounding nerve fibers (arrows). (f) PF x10: moderate, patchy, lymphoplasmacytic ventriculitis. (g) PF x20 and (h) PF x10: detail of lymphoplasmacytic perivascular cuffing with adjacent vasogenic edema and gliosis with rare gemistocytes (arrow). (i) PF x40: detail of the leptomeningeal lymphoplasmacytic and histiocytic infiltrates with sparse numbers of neutrophils (arrow).

**Figure:**
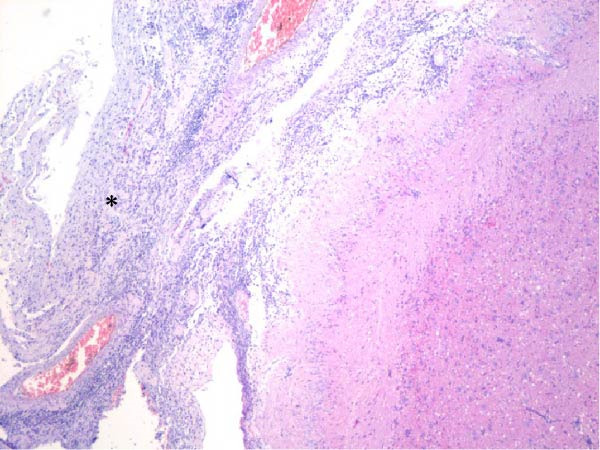
(a)

**Figure:**
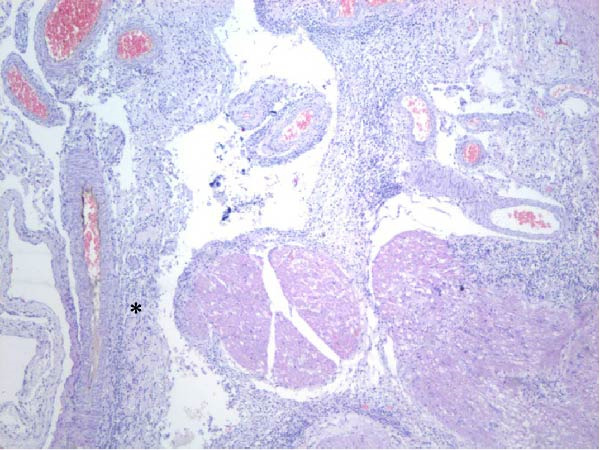
(b)

**Figure:**
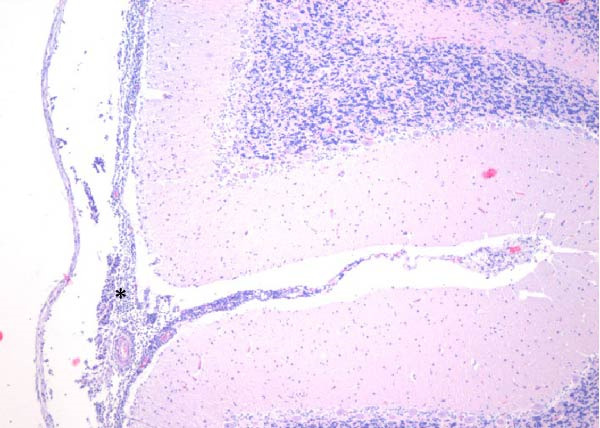
(c)

**Figure:**
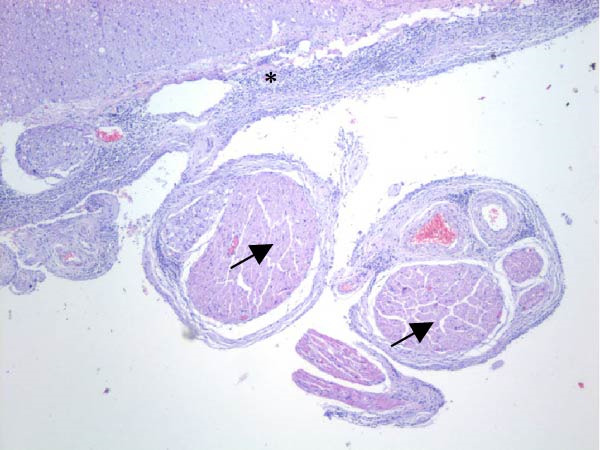
(d)

**Figure:**
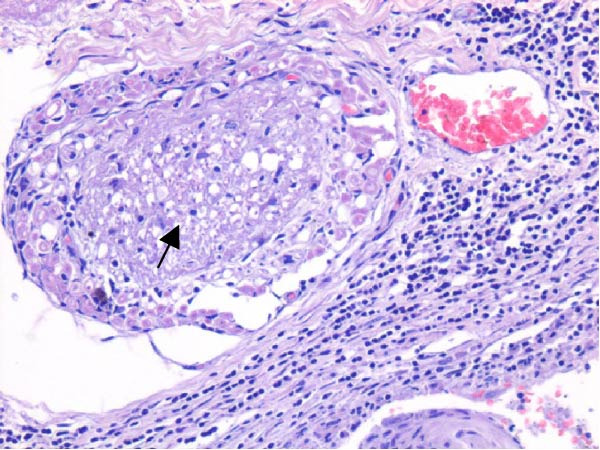
(e)

**Figure:**
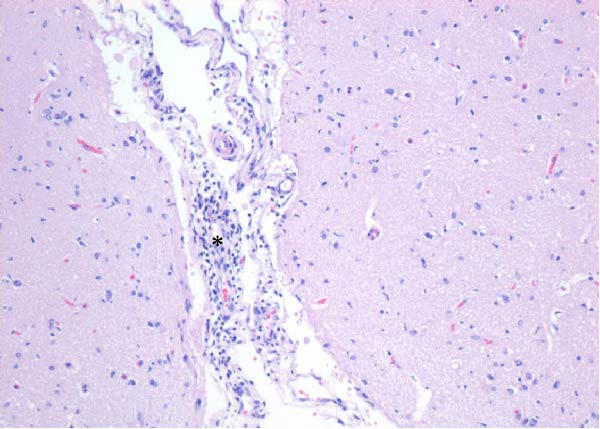
(f)

**Figure:**
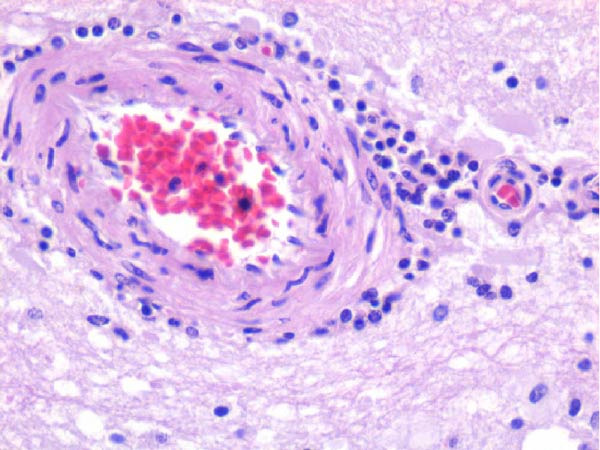
(g)

**Figure:**
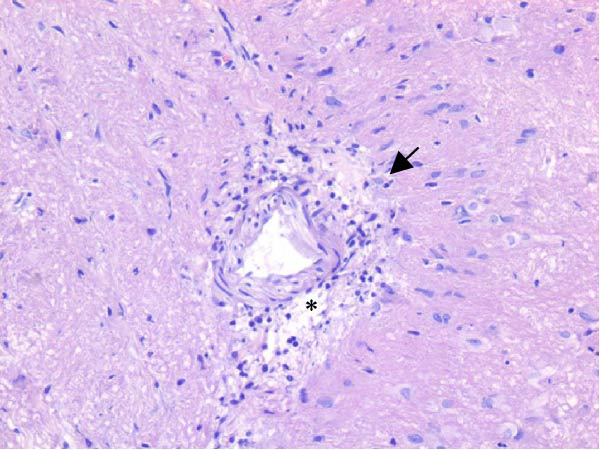
(h)

**Figure:**
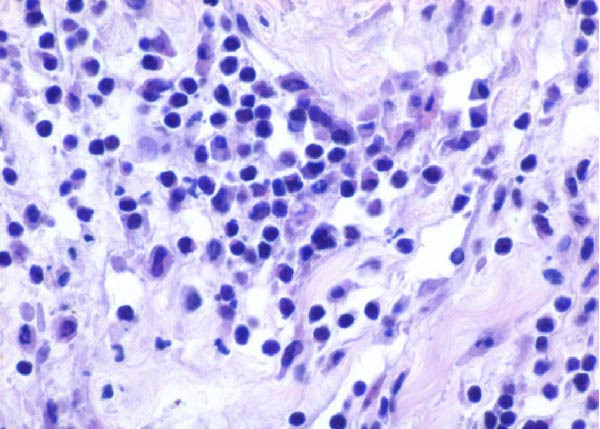
(i)

**Table 2:** Neuropathological alterations.

ID	Brain								Associated CNS lesions	Detection of *Brucella*	Strain	Coinfection	Causative etiology other than *Brucella*
	Me	PC	G	Type of inflammatory infiltrate	NN	M	H	D					
Sc 18.06.11	+++	+++	−	Lymphoplasmacytic	−	−	−	Diffuse	Lymphoplasmacytic and histioctytic meningomyelitis and polyradiculoneuritis	Culture, RT PCR, ELISA, and RBT	ST26	AHV [76]	—
Sc 07.07.11	++	−	+	Lymphoplasmacytic	−	−	−	Multifocal	Lymphoplasmacytic meningitis	Culture, RT PCR ELISA, and RBT	ST26	GHV [76]	—
Sc 29.07.11	−	−	+	−	−	−	−	Multifocal	−	Culture and ELISA	ST49	CeMV [77]	—
Sc 20.10.12	−	++	++	Lymphoplasmacytic	++	+	−	Multifocal	Lymphoplasmacytic myelitis	−	−	−	CeMV
Gg 29.03.13	−	−	−	−	−	−	−	−	−	−	−	GHV and AHV [76]	—
Sc 25.10.13	+++	+++	++	Lymphoplasmacytic and histiocytic with few neutrophils	+	−	−	Diffuse	Lymphoplasmacytic meningomyelitis	Culture, RT PCR, ELISA, and RBT	ST26	−	—
Sc 13.07.14	−	++	++	Lymphoplasmacytic	+	+	−	Multifocal	−	−	−	−	—
Sc 15.08.14	++	NA	NA	NA	NA	NA	NA	NA	−	Culture, RT PCR, ELISA, and RBT	ST26	−	—
Sc 13.02.15	−	+	−	−	−	−	−	Multifocal	−	−	−	−	—
Sc 20.03.15	−	−	−	−	−	−	−	−	−	RT PCR	−	−	—
Sc 19.08.15	+++	+++	++	Lymphoplasmacytic and histiocytic with few neutrophils	−	−	−	Diffuse	−	Culture and RT PCR	ST49	−	—
Gg 22.09.15	−	+	+++	Lymphocytes and gemistocytes	−	++	−	Focal	−	−	−	−	—
Gg 30.03.16	−	−	−	−	−	++	+++	−	−	−	−	−	—
Sc 31.07.16	+++	+	++	Lymphoplasmacytic and histiocytic with few neutrophils	−	−	−	Diffuse	−	−	−	−	—
Sc 10.10.16	−	+	−	Lymphoplasmacytic	−	−	−	Focal	−	RT PCR	−	−	—
Sc 19.12.16	−	−	−	−	−	−	−	−	−	RT PCR	−	−	—
Sc 20.02.17	−	+	+	Lymphoplasmacytic	+	−	−	Multifocal	−	−	−	−	—
Sc 19.04.17	+++	+++	++	Lymphoplasmacytic and histiocytic with few neutrophils	−	−	−	Diffuse	Lymphoplasmacytic and histioctytic meningomyelitis and polyradiculoneuritis	Culture and RT PCR	ST49	−	—
Tt 28.04.18	−	−	−	−	−	−	−	−	−	−	−	−	—
Sc 26.07.19	+++	++	++	Lymphoplasmacytic and histiocytic with few neutrophils	−	−	−	Diffuse	−	RT PCR	−	−	—
Sc 01.03.21	+++	+++	+	Lymphoplasmacytic and histiocytic with few neutrophils	−	−	−	Diffuse	Lymphoplasmacytic and histioctytic meningomyelitis and polyradiculoneuritis	Culture and RT PCR	ST26	−	—
Sc 04.05.21	+	++	+	Lymphoplasmacytic and histiocytic with few neutrophils	−	−	−	Multifocal	Lymphoplasmacytic and histioctytic meningomyelitis	Culture and RT PCR	ST49	−	—
Sc 06.08.21	+++	+	+	Lymphoplasmacytic and histiocytic with few neutrophils	−	−	−	Multifocal	−	Culture and RT PCR	ST49	GHV [78]	—

*Note:* Meningitis (Me), perivascular cuffing (PC), gliosis (G), neuronal necrosis (NN), malacia (Ma), hemorrhage (H), and distribution (D). The following lesions in brain and spinal cord were pondered and scored as follows: the cellular infiltration was indicated as + for scarce, ++ for moderate, +++ for abundant, and − for absent. Gliosis was denoted with + for presence and − for absence. Perivascular cuffs were indicated with − for absence, + for presence, and in parentheses, the type.

Abbreviations: AHV, alphaherpesvirus; CeMV, cetacean morbillivirus; CNS, central nervous system; GHV, gammaherpesvirus; lymph, lymphoplasmacitic.

**Figure 2:**
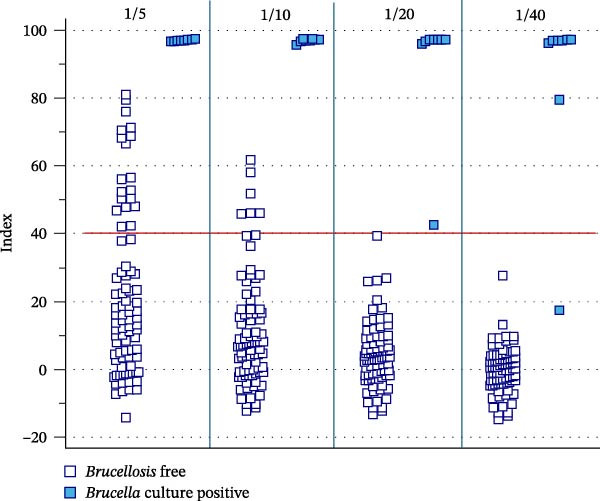
Inhibition percentage distribution in dolphin sera across serum dilutions. Distribution of the inhibition percentage values (Index) obtained when testing sera from 84 brucellosis free (white squares) and 8 *Brucella* culture positive (blue squares) dolphins in a commercial blocking ELISA (I.B Compac) using different serum dilution (from 1/5 to 1/40). The continuous horizontal line represents the cut‐off (40%) recommended by the manufacturer.

Regarding serological analysis, when the gold‐standard sera were run in the commercial bELISA (Figure 2), we observed that using the serum dilutions recommended by the manufacturer for ruminants and pigs (1/5 and 1/10) led to a marked loss of specificity. While the positive control group was correctly classified above the manufacturer cut‐off (40%), 19 and six false positives were detected in the Brucellosis‐free population at a 1/5 and 1/10 dilution, respectively. When we adjusted the serum dilution (1/20 and 1/40) and maintained the 40% cut‐off to achieve 100% diagnostic specificity (i.e., no false positives in the Brucellosis‐free population), all the eight culture‐positive cetaceans remained positive at 1/20 dilution, while one of them fell into the negative range at 1/40. In the RBT, four of these sera tested also positive, two of them were negative, and the remaining two could not be tested because of the high degree of hemolysis.

### 3.2. Genetic Study

**Figure 3:**
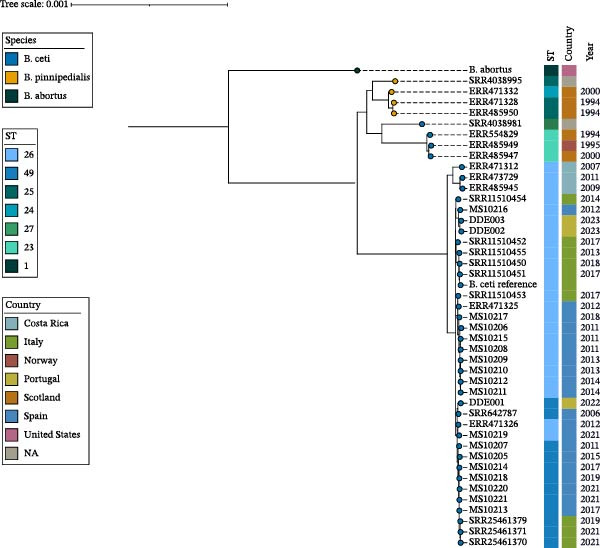
Maximum‐likelihood phylogenetic tree. Strains sequenced in the present study are MS10205‐MS10220. External sequences of *Brucella* are named according to European Nucleotide Archive or Sequence Read Archive identifier. *Brucella* ST, *Brucella* species, country, and year of identification are indicated in the four bars on the right.

In the constructed phylogenetic tree (Figure 3), three primary clades were evident, distinguishing *B. pinnipedialis* (ST24 and ST25), *B. ceti* belonging to ST23 and ST27, and *B. ceti* belonging to ST26 and ST49. All the 17 isolates examined in this study clustered with *B. ceti* isolates belonging to ST26 (*n* = 10) and ST49 (*n* = 7) and exhibited a difference of 57–148 SNPs between the two clusters. Furthermore, these isolates were located in the same clade with other sequences that were previously detected in Spain, along with strains that were isolated in Italy and Portugal, during the same period in which isolates from this study were retrieved. On the other hand, the analysis of SNP differences between isolates from the same individual (*n* = 3) revealed that, in two individuals, identical isolates were retrieved from different tissues (no hqSNP detected), while five hqSNPs were found differentiating isolates from the third individual (Table 3). In addition, the comparison between isolates from different individuals showed in some cases 2–10 hqSNPs between sequences (Table 3).

**Table 3:** SNP differences between isolates.

Animal ID	Strain ID	Stranding year	Tissue sample	ST	hqSNPs
Sc 25.10.13	MS10209	2013	Cerebrum	ST26	0
	MS10210	2013	Tracheobronchial lymph node	ST26	
Sc 15.08.14	MS10211	2014	Cerebrum	ST26	0
	MS10212	2014	Spleen	ST26	
Sc 19.04.17	MS10213	2017	Tracheobronchial lymph node	ST49	5
	MS10214	2017	Cerebrum	ST49	
Sc 07.07.11	MS10208	2011	Cerebrum	ST26	2
Sc 25.03.11	MS10215	2011	Cerebrum	ST26	
Sc 19.04.17	MS10214	2017	Cerebrum	ST49	5
Sc 01.08.19	MS10218	2019	Cerebrum	ST49	
Sc 19.04.17	MS10213	2017	Tracheobronchial lymph node	ST49	4
Sc 01.08.19	MS10218	2019	Brain	ST49	
Sc 25.03.11	MS10215	2011	Cerebrum	ST26	144
Sc 01.08.17	MS10216	2017	Cerebrospinal fluid	ST26	
Sc 04.05.21	MS10220	2021	Tracheobronchial lymph node	ST49	10
Sc 06.08.21	MS10221	2021	Cerebrospinal fluid	ST49	

## 4. Discussion

Estimating the prevalence of brucellosis in cetaceans is particularly challenging due to methodological limitations, including difficulties in obtaining high‐quality samples and the absence of properly validated indirect diagnostic tools [6]. A recent culture‐based study of stranded dolphins from the Spanish northwestern Mediterranean reported a culture‐based prevalence of 7.3% [79], whereas seroprevalence data obtained from nonvalidated tests suggest higher values. Here, we present an extensive study of *B. ceti* infection in dolphins from the Spanish Mediterranean coast, with the dual aim of assessing diagnostic approaches for epidemiological surveillance and contributing to the phylogenetic understanding of this infection. This comprehensive investigation involved the analysis of 30 brucellosis suspicious dolphins stranded between 2011 and 2021 and the genetic characterization of *B. ceti* strains isolated in 14 of them.

In brucellosis, bacterial isolation is considered the gold‐standard and the only unequivocal method to confirm an outbreak. However, culture is impractical for surveillance purposes; thus, immunological and molecular tests are needed to approximate the diagnosis [65]. In our study, brucellosis was confirmed by bacterial culture in 14/30 individuals (46.7%), while presence of *Brucella* spp. DNA was detected in 15/30 (50.0%) using a direct qPCR and the subsequent sequencing of DNA products. While direct qPCR detected most culture‐positive cases (PPV = 73%), some discrepancies remained. It is worth noting that in the four qPCR–positive/culture‐negative cases, only a single sample (cerebellum, amniotic fluid, or spleen) tested positive, and only one of these was available for culture (Supporting Information 2: Table S2). Consequently, it remains unclear whether these discrepancies reflect specificity issues or superior analytical sensitivity in qPCR. Interestingly, in the three culture‐positive/qPCR–negative dolphins, *B. ceti* was isolated from samples that had tested negative by qPCR, suggesting that, in these instances, culture was more effective at detecting the infection. Overall, the rate of positive samples was higher in bacterial culture compared to qPCR, particularly in the CNS. In contrast, other authors based the diagnosis primarily on direct PCR and serology, since culture attempts were unsuccessful [44]. These contrasting findings likely reflect methodological flaws or tissue sample degradation, as previously suggested [80]. Regarding the most diagnostically informative tissues, infection was predominantly detected in the CNS by both qPCR and bacterial culture, consistent with the neurotropism attributed to *B. ceti* in cetaceans, especially in striped dolphins [6, 20, 23, 43, 81]. *Brucella* was most frequently isolated from the cerebrum (*n* = 8) and CSF (*n* = 5) (see Supporting Information 2: Table S2), which should be prioritized when assessing neurobrucellosis in cetaceans. Our results also underscore the diagnostic value of reproductive tissues, spleen, and other lymphoid organs for molecular or cultured‐based detection, supporting previous observations [43] and highlighting their usefulness as target samples for brucellosis surveillance in marine mammals.

Overall, our results indicate that, with appropriate protocols, selective media, and optimized sampling, bacterial culture can match or even surpass the diagnostic sensitivity of direct molecular methods. While culture remains essential for confirming infection and enabling phenotypic and genotypic analyses, a well‐validated qPCR [64] allows rapid detection and identification of *Brucella* DNA, thereby supporting diagnostics and surveillance efforts.

Serological diagnosis in our study was limited by the short availability of high‐quality sera. Nonetheless, we used a small panel of gold‐standard sera from culture‐positive dolphins and a larger collection from Brucellosis‐free populations to evaluate the performance of the commercial bELISA, originally designed for terrestrial animals, for diagnosing brucellosis in dolphins. Commercial blocking or competitive ELISA kits have been proposed for serological studies of *Brucella* in cetaceans and other marine mammal species [22, 44, 46, 47, 79], owing to their lack of species specificity and tolerance for suboptimal samples [46]. However, since gold‐standard sera from marine mammals are rarely available, these tests remain largely unvalidated [45]. The bELISA used in our study was also applied in a recent work, in which the authors did not validate the test but titrated sera from dolphins with uncertain health status [79]. Although our sample size was insufficient to provide robust estimates of diagnostic sensitivity and specificity for this bELISA, we started from the manufacturer‐recommended serum dilutions (1:5 for ovine/caprine and 1:10 for bovine/camelid/porcine samples) and included additional serial dilutions (1:20 and 1:40) to better assess test performance in dolphins. Preliminary results indicated that the lower dilutions recommended by the manufacturer could compromise specificity in this species, highlighting the importance of optimizing serum dilution and validating the assay before broader application in marine mammals. According to our results, a serum dilution of 1:20 combined with a cut‐off corresponding to the 1:40 dilution could be potentially recommended; however, confirmation with larger panels of gold‐standard sera is needed to establish definitive dilution and cut‐off values.

On the other hand, the RBT failed to detect antibodies in two culture and bELISA‐positive animals and could not be performed in hemolyzed sera. In various studies conducted with stranded cetaceans from different geographic areas, negative results have been obtained in the RBT, despite the successful isolation of the bacteria [20, 22, 43], indicating that the RBT test, while practical for rapid serological screening, may produce false negative results due to a variety of factors [82].

The extensive lymphoplasmacytic and histiocytic meningoencephalomyelitis associated with *Brucella* spp. in the present case series was consistent with previously reported neurobrucellosis in cetaceans [16, 83]. Perivascular cuffing and polyradiculoneuritis were also hallmark features. All together these histopathological features were unique amongst the inflammatory responses in the CNS associated with other common neurotropic etiologies in cetaceans such as CeMV, herpesviruses, *Toxoplasma gondii*, *Nasitrema* sp., or bacterial septicaemias [16, 83]. Both CeMV and herpesviruses tend to induce nonsuppurative meningoencephalitis and perivascular cuffing [16, 84]. However, the inflammatory infiltrates, particularly within the meninges, are much less dense and predominantly consistent of lymphocytes and plasma cells, while the additional presence of histiocytes in equal proportions with the previous, together with neutrophils, has proven to be useful diagnostic tools for cetacean neurobrucellosis. Interestingly, human neurobrucellosis holds a similar histologic pattern [16, 23, 83, 85]. *Toxoplasma gondii* multifocal inflammatory infiltrates in the neuropil with cysts, while *Nasitrema* sp. and other aberrant parasite migrations induce multifocal granulomatous reactions with malacia [16, 86].

Three of the present cases were exceptions regarding the histological pattern. One animal with concomitant CeMV and *B. ceti* infection showed only mild multifocal gliosis, which suggested the possibility of a subclinical infection, similar to what occur in human neurobrucellosis [87]. As for CeMV, this pattern has also been reported for chronic and/or subclinical infections, particularly in animals detected between epidemics [88]. *B. ceti* was detected by RT PCR only in the amniotic fluid from a striped dolphin in the absence of lesions. The third exception was a striped dolphin with severe meningoencephalitis matching the pattern for neurobrucellosis but negative all *Brucella* spp. trials, with no available results for other neurotropic etiologies from this or other studies. This case raises the question on the existence of other agents with neurotropism in cetaceans with similar pathogenesis and lesional pattern as *Brucella* spp.

A notable observation from these case series is that, although evaluating coinfections was not the primary aim of this study, some individuals had previously been tested for other pathogens in independent investigations, the results of which have been published previously [76–78, 89], and multiple coinfections were detected, including GHV in three individuals (Sc.07.07.11, Sc 19.12.11, and Sc 06.08.21); GHV and CeMV in two animals (Sc 29.07.11 and Sc 20.10.12); AHV, GHV, and CeMV in one animal (Sc 25.03.11); and AHV in another animal (Sc 18.06.11). These concomitant infections, therefore, could play a pathogenic role in CNS lesions. Coinfections between *Brucella* spp. and other pathogens in cetaceans have been previously reported in different parts of the world [21, 32, 90], including in the Mediterranean Sea [20, 91, 92]. Furthermore, other agents, such as CeMV, herpesvirus, or toxoplasmosis, cause neuropathological changes and inflammation that must be differentiated from the *Brucella* spp. neuroinflammatory pattern [16, 93]. In the case of the Sc 20.10.12 animal, an infection with CeMV was previously confirmed [77], and this was the only positive case for *B. ceti* with mild gliosis as the only lesion in CNS. On the other hand, in the case of the Sc 13.07.14 animal, although confirmation of infection with this virus was not possible, CNS lesions and interstitial bronchopneumonia compatible with CeMV were observed, suggesting a potential association of the lesions with this agent. Additionally, the animal Gg 29.03.13 was infected with both GHV and AHV and negative in all trials for *B. ceti* [76]. This animal lacked CNS lesions of any kind.

Regarding bacterial characterization, it is interesting that of the five cases in which the bacteria were cultured, sequenced, and their genomes characterized, four were associated with ST49, and one with ST26. However, given the very small sample size (*n* = 5), it is important to interpret these results with caution and acknowledge the need for further research with larger samples. In a recent study, no significant pathological differences were found between ST49 and ST26 similar to what was observed in the present study; however, that study also emphasized the limitation of its small sample size [43].

In our study, *B. ceti* was found exclusively in striped dolphins, further supporting the hypothesis, well established in the literature, that this species is a particularly susceptible host [6, 12, 14, 18, 20, 23, 24, 32, 43, 81, 94–96]. In contrast, none of the bottlenose dolphins or Risso’s dolphins analyzed tested positive. This is particularly notable in the case of bottlenose dolphins, a species in which brucellosis has been widely reported across multiple regions [11, 22, 44, 97–99]. The absence of positive cases in our sample may reflect regional variation in prevalence or temporal factors. While *B. ceti* has occasionally been reported in Risso’s dolphins [100], other studies have not found evidence of brucellosis in this species [79, 101]. These findings may reflect interspecies differences in susceptibility but could also be influenced by ecological, behavioral, or epidemiological factors affecting exposure and transmission dynamics among cetacean populations. Our observations, including some presumptive cases, may help guide future studies aimed at clarifying *Brucella* epidemiology in these species. Regarding the phylogenetic analysis, classically, *B. ceti* has been divided into three STs: ST23, associated with isolates from porpoises; ST26, associated with isolates from dolphins; and ST27, associated with isolates from both common bottlenose dolphins and humans [6–10]. Among these, ST27 is the only one confirmed as zoonotic [102, 103]. However, some genetic studies suggest that ST27 may not fully align with the classification of either *B. ceti* or *B. pinnipedialis* [8, 11]. Recently, a new ST, ST49, was found in three stranded striped dolphins from Italy [43], common dolphins (*Delphinus delphis*) from Portugal [80], and in one dolphin (species unspecified) from Spain [68]. Our study identified eight cases involving ST26 and six cases involving the recently described ST49, confirming the circulation of both STs within the Mediterranean Sea [20, 43]. These findings further support the inclusion of ST49 as a ST within the species *B. ceti*, alongside the traditionally recognized STs. Notably, no sequences associated with the zoonotic ST27, though its presence has been reported in cetaceans from Mediterranean waters, particularly along the coast of Croatia [48, 98]. This aligns with studies conducted in Italy, where strains of ST27 were also absent [20, 43]. On the other hand, the fact that all sequences described in this study were found within the same clade as other sequences detected in Spain, and Italy supports the previously suggested association between phylogeny and geographical distribution [20, 43].

The comparison of SNP differences between *B. ceti* strains isolated from different organs of the same individual can provide valuable insights into the dynamics of *Brucella* infection in cetaceans and its potential adaptation to various tissues or organs. Furthermore, this information, along with the comparison of bacterial genomes from different individuals, represents valuable data that may aid in establishing SNP thresholds for distinguishing epidemiologically related strains in the future, as previously suggested [22]. Our results (Table 3) revealed that variation in the number of hqSNPs from isolates retrieved from a single individual could vary from none (two animals infected with ST26 strains) to three (one animal infected with a ST49 strain). Notably, little disparity of hqSNPs was also observed when comparing two genomes from cetaceans stranded 3.5 months apart (two hqSNPs) and more than 2 years apart (four and five hqSNPs). In other cases, the comparison of genome sequences from cetaceans stranded with a 3‐month difference revealed a difference of 10 hqSNPs (Table 3). These findings highlight the limited variability among some of the strains isolated from individuals in different years, suggesting that the infection in these animals might have been caused by strains belonging to the same lineage and thus could be potentially related epidemiologically. Conversely, other strains originating from cetaceans stranded over 6 years apart exhibited a significantly higher SNP count (144 hqSNPs). As far as we know, the sole dataset available for comparative analysis concerning marine brucellas with a likely epidemiological link consists of two strains of *B. pinnipedialis*, presumably transmitted from a female bottlenose dolphin to her offspring that differed by 18 hqSNPs [22]. These observations emphasize the significance of comprehensive genomic research on *Brucella* within the context of marine mammal infections to enhance our understanding of its epidemiology and evolutionary dynamics. Further research should be conducted to increase our knowledge on a SNP threshold to differentiate epidemiologically related *Brucella* strains as previously suggested for other *Brucella* species like *B. melitensis* [104].

An additional observation is that in cases where strains from diverse anatomical locations have been subjected to WGS and phylogenetic study, they consistently reveal infection by the same ST. This contrasts with the findings of a North American cetacean study, where coinfections involving multiple STs were observed in 11 animals [99].

In conclusion, this research work confirms the neurotropism of *B. ceti* and high susceptibility of striped dolphins and reports the circulation of ST26 and the emerging ST49 in the western Mediterranean. Regarding diagnosis, we highlight the need for proper validation of serological tests (particularly commercial bELISA) and show that, with optimized protocols, bacterial culture is highly effective for confirmatory purposes, while histopathology and qPCR provide useful tools to support surveillance.

**Nomenclature**
ST: Sequence type
WGS: Whole‐genome sequencing
SNP: Single nucleotide polymorphism
hqSNPs: High‐quality SNPs
RBT: Rose Bengal test
bELISA: Blocking ELISA
H&E: Hematoxylin and eosin
CSF: Cerebrospinal fluid
PBS: Phosphate‐buffered saline
PPV: Positive predictive values
NPV: Negative predictive values
OD_450 nm_: Optical density
Ct: Cycle threshold
CNS: Central nervous system
GHV: Gammaherpesvirus
AHV: Alphaherpesvirus
CeMV: Cetacean morbillivirus.

## Author Contributions

Conceptualization: Ignacio Vargas‐Castro, Sara Andrés‐Barranco, José Luis Crespo‐Picazo, Mª Ángeles Jiménez‐Martínez, Pilar María Muñoz, María Jesús de Miguel, and Daniel García‐Párraga. Methodology: Ignacio Vargas‐Castro, Sara Andrés‐Barranco, José Luis Crespo‐Picazo, Laura Torre‐Fuentes, Mª Ángeles Jiménez‐Martínez, Marta Hernández, Manuel Arbelo, Julio Álvarez, Pilar María Muñoz, Vicente Marco‐Cabedo, María Jesús de Miguel, Débora López, and Marta Muñoz‐Baquero. Formal analysis: Laura Torre‐Fuentes, Marta Hernández, and Julio Álvarez. Investigation: Ignacio Vargas‐Castro, Sara Andrés‐Barranco, José Luis Crespo‐Picazo, Mª Ángeles Jiménez‐Martínez, Pilar María Muñoz, and María Jesús de Miguel. Writing – original draft preparation: Ignacio Vargas‐Castro, José Luis Crespo‐Picazo, and Mª Ángeles Jiménez‐Martínez. Writing – review and editing: Ignacio Vargas‐Castro, Sara Andrés‐Barranco, José Luis Crespo‐Picazo, Laura Torre‐Fuentes, Mª Ángeles Jiménez‐Martínez, Marta Hernández, Manuel Arbelo, Julio Álvarez, Pilar María Muñoz, Vicente Marco‐Cabedo, María Jesús de Miguel, Débora López, Marta Muñoz‐Baquero, Daniel García‐Párraga, and José Ángel Barasona. Supervision: Mª Ángeles Jiménez‐Martínez, Pilar María Muñoz, Daniel García‐Párraga, and José Ángel Barasona. Funding acquisition: Pilar María Muñoz, Daniel García‐Párraga, and José Ángel Barasona.

## Funding

This work was supported by a collaborative agreement involving the Fundación Oceanogràfic and the VISAVET Center of Complutense University of Madrid. CITA work was supported by Aragon Government (Grupo de Investigación A21_23R). José A. Barasona is a recipient of a “Ramón y Cajal” contract (RYC2022‐038060‐I) funded by the Spanish Ministry of Science and Innovation (MCIN/AEI) and Fondo Social Europeo Plus (FSE+).

## Disclosure

All authors have read and approved the final version of the manuscript to be published.

## Conflicts of Interest

The authors declare no conflicts of interest.

## Supporting Information

Additional supporting information can be found online in the Supporting Information section.

## Supporting information


**Supporting Information 1** Table S1. Correspondence between the ID of each isolate and the ID of the animal to which it belongs (composed of species and stranding date dd.mm.yyyy). ST: sequence type.

**Supporting Information 2** Table S2. Laboratory diagnosis of Brucella spp.: culture, qPCR, and serology (RBT and blocking ELISA). Animals are identified by the initial letters of the Latin species (e.g., Stenella coeruleoalba: Sc, Tursiops truncatus: Tt; Grampus griseus: Gg) and the stranding date (day.month.year). The animals were arranged chronologically based on the year of stranding. Positive results in culture, real‐time PCR, and ELISA are highlighted in bold and underlined. The Ct values of positive samples in the real‐time PCR are indicated in parentheses. Results of the RBT are indicated with + (positive) and − (negative). ST: sequence type; F: female; M: male; A: adult, J: juvenile; NA: not available.

**Supporting Information 3** Table S3. Data of the sequences included in the phylogenetic study.

## Data Availability

The datasets generated during the current study are available in the European Nucleotide Archive (PRJEB96491).
