# Supplementary material for: Towards Integrated Surveillance of Marine Brucellosis: Diagnostic and Phylogenetic Assessment of Brucella ceti in Stranded Dolphins of the Western Mediterranean Sea
Source: Transbound Emerg Dis. 2026 Jan 31;2026:2075116. doi: 10.1155/tbed/2075116 (PMC12859529; doi:10.1155/tbed/2075116)
Supplement: Supplementary file 1 — Supporting Information 1 Table S1. Correspondence between the ID of each isolate and the ID of the animal to which it belongs (composed of species and stranding date dd.mm.yyyy). ST: sequence type. [file TBED-2026-2075116-s001.docx]

**Supplementary Table S1. Correspondence between the ID of each isolate and the ID of the animal to which it belongs** (composed of species and stranding date dd.mm.yyyy). ST: Sequence Type.

| **ID isolate** | **ID animal** | **ST** |
| --- | --- | --- |
| MS10205 | Sc 19.08.15 | 49 |
| MS10206 | Sc 18.06.11 | 26 |
| MS10207 | Sc 29.07.11 | 49 |
| MS10208 | Sc 07.07.11 | 26 |
| MS10209 | Sc 25.10.13 | 26 |
| MS10210 | Sc 25.10.13 | 26 |
| MS10211 | Sc 15.08.14 | 26 |
| MS10212 | Sc 15.08.14 | 26 |
| MS10213 | Sc 19.04.17 | 49 |
| MS10214 | Sc 19.04.17 | 49 |
| MS10215 | Sc 25.03.11 | 26 |
| MS10216 | Sc 01.08.17 | 26 |
| MS10217 | Sc 26.04.18 | 26 |
| MS10218 | Sc 01.08.19 | 49 |
| MS10219 | Sc 01.03.21 | 26 |
| MS10220 | Sc 04.05.21 | 49 |
| MS10221 | Sc 06.08.21 | 49 |
