# Supplementary material for: Towards Integrated Surveillance of Marine Brucellosis: Diagnostic and Phylogenetic Assessment of Brucella ceti in Stranded Dolphins of the Western Mediterranean Sea
Source: Transbound Emerg Dis. 2026 Jan 31;2026:2075116. doi: 10.1155/tbed/2075116 (PMC12859529; doi:10.1155/tbed/2075116)
Supplement: Supplementary file 2 — Supporting Information 2 Table S2. Laboratory diagnosis of Brucella spp.: culture, qPCR, and serology (RBT and blocking ELISA). Animals are identified by the initial letters of the Latin species (e.g., Stenella coeruleoalba: Sc, Tursiops truncatus: Tt; Grampus griseus: Gg) and the stranding date (day.month.year). The animals were arranged chronologically based on the year of stranding. Positive results in culture, real‐time PCR, and ELISA are highlighted in bold and underlined. The Ct values of positive samples in the real‐time PCR are indicated in parentheses. Results of the RBT are indicated with + (positive) and − (negative). ST: sequence type; F: female; M: male; A: adult, J: juvenile; NA: not available. [file TBED-2026-2075116-s002.docx]

**Supplementary Table S2. Laboratory diagnosis of *Brucella* spp.: culture, qPCR and serology (RBT and blocking ELISA).** Animals are identified by the initial letters of the Latin species (e.g., *Stenella* *coeruleoalba*: Sc, *Tursiops truncatus*: Tt; *Grampus griseus*: Gg) and the stranding date (day.month.year). The animals were arranged chronologically based on the year of stranding. Positive results in culture, Real-Time PCR, and ELISA are highlighted in bold and underlined. The Ct values of positive samples in the Real-Time PCR are indicated in parentheses. Results of the RBT are indicated with + (positive) and - (negative). ST: Sequence Type; F: Female; M: Male; A: Adult, J: Juvenile; NA: Not available

| **ID** | **Sex** | **Age** | ***Brucella* spp. culture** | **qPCR** | **RBT** | **Blocking ELISA (%PI)**  **Serum dilution 1/20 and cutoff >40%** |
| --- | --- | --- | --- | --- | --- | --- |
| **Sc25.03.11** | F | J | Cerebrum, **cerebellum**, tracheobronchial lymph node, spleen | Cerebrum, cerebellum, pharyngeal tonsils, lung, tracheobronchial lymph node, liver, spleen, blood | - | **97,20** |
| **Sc18.06.11** | M | J | Cerebrum, pharyngeal tonsils, tracheobronchial lymph node, **spleen** | **Cerebrum (35.66)**, pharyngeal tonsils, lung, **tracheobronchial lymph node (36.54)**, liver, spleen | + | **96,87** |
| **Sc07.07.11** | M | J | **Cerebrum**, pharyngeal tonsils, tracheobronchial lymph node, **spleen** | Cerebrum, pons, **thalamus (35.46)**, pharyngeal tonsils, lung, tracheobronchial lymph node, liver, spleen | + | **97,23** |
| **Sc29.07.11** | F | A | Cerebrum, pharyngeal tonsils, tracheobronchial lymph node, **spleen** | Cerebrum, pons, cerebellum, lung, liver, spleen | - | **96,01** |
| **Sc19.12.11** | F | C | Cerebrum, pharyngeal tonsils, tracheobronchial lymph node, spleen | Cerebrum, pons, thalamus, cerebellum, meninges, pharyngeal tonsils, lung, tracheobronchial lymph node, liver, spleen | - | 6,47 |
| **Sc20.10.12** | M | J | Cerebrum, pharyngeal tonsils, lung, pulmonary exudate, tracheobronchial lymph node, spleen | Pons, thalamus, lateral ventricle, cerebellum, pharyngeal tonsils, lung, tracheobronchial lymph node, liver, testis | NA | NA |
| **Gg29.03.13** | F | J | Cerebrum, pharyngeal tonsils, tracheobronchial lymph node, spleen | Cerebrum, pons, thalamus, lateral ventricle, cerebellum, spinal cord, pharyngeal tonsils, lung, tracheobronchial lymph node, liver, spleen, blood | NA | NA |
| **Sc25.10.13** | F | A | **Cerebrum**, pharyngeal tonsils, **tracheobronchial lymph node**, **spleen** | **Cerebrum (34.58)**, **pons (31.28)**, **lateral ventricle (35.02)**, cerebellum, **spinal cord (32.85)**, pharyngeal tonsils, lung, tracheobronchial lymph node, liver, spleen, uterus | + | **97,18** |
| **Sc13.07.14** | M | A | Cerebrum, lung, tracheobronchial lymph node, spleen, urinary bladder, penis | Cerebrum, pons, cerebellum, pharyngeal tonsils, lung, bronchial secretion, tracheobronchial lymph node, spleen | NA | NA |
| **Sc15.08.14** | F | A | **Cerebrum**, **pharyngeal tonsils**, tracheobronchial lymph node, **spleen**, ovary | **Cerebrum (31.99)**, **pons (35.31)**, **cerebellum (34.01)**, **spinal cord (32.32)**, pharyngeal tonsils, lung, tracheobronchial lymph node, liver spleen, **mammary gland (33.24)** | + | **97,26** |
| **Sc13.02.15** | M | C | Cerebrum, pharyngeal tonsils, lung, tracheobronchial lymph node, penis | Pons, cerebellum, lung, liver | NA | NA |
| **Sc20.03.15** | F | A | Cerebrum, tracheobronchial lymph node, spleen, pharyngeal tonsils, ovary | Cerebrum, pons, thalamus, cerebellum, spinal cord, pharyngeal tonsils, lung, tracheobronchial lymph node, liver, spleen, umbilical cord, **amniotic fluid (37.04)**, cerebrum (fetus), liver (fetus), blood | NA | NA |
| **Sc19.08.15** | M | A | **Cerebrum**, **CSF**, tracheobronchial lymph node, epididymis | **Lateral ventricle (33.86)**, pharyngeal tonsils, lung, tracheobronchial lymph node, liver, spleen, epididymis | NA | NA |
| **Gg22.09.15** | M | A | Cerebrum, medullary and ventricular CSF, tracheobronchial lymph node, testis, urine | Cerebrum, cerebellum, spinal cord, pharyngeal tonsils, lung, tracheobronchial lymph node, liver, spleen, testis, epididymis | NA | NA |
| **Gg30.03.16** | F | A | Cerebrum, CSF, lung, urinary bladder, urine, uterus, amniotic fluid, umbilical cord | Cerebrum, spinal cord, pharyngeal tonsils, lung, tracheobronchial lymph node, liver, spleen, uterus | NA | NA |
| **Sc09.07.16** | M | A | Cerebrum, tracheobronchial lymph node, spleen, pharyngeal tonsils | Lung, liver | NA | NA |
| **Sc31.07.16** | F | A | Cerebrum, CSF, pharyngeal tonsils, lung, tracheobronchial lymph node, spleen, kidney, urinary bladder, ovary, uterus, mammary gland | Cerebrum, cerebellum, CSF, pharyngeal tonsils, lung, liver, spleen, blood | NA | NA |
| **Sc10.10.16** | M | A | Cerebrum, lung, tracheobronchial lymph node, spleen, kidney, testis | Cerebrum, lung, liver, **spleen (33.31)**, testis | NA | NA |
| **Sc19.12.16** | F | A | Cerebrum, lung, tracheobronchial lymph node, spleen | **Cerebellum (39.18)**, spinal cord, lung, liver, spleen | NA | NA |
| **Sc20.02.17** | M | A | Cerebrum, lung, tracheobronchial lymph node, spleen, kidney | Cerebrum, cerebellum, spinal cord, lung, liver, spleen | NA | NA |
| **Sc19.04.17** | F | J | **Cerebrum**, **CSF**, pharyngeal tonsils, **tracheobronchial lymph node**, **spleen** | Cerebrum, **cerebellum (34.49)**, **spinal cord (35.49)**, pharyngeal tonsils, lung, tracheobronchial lymph node, **liver (35.53)**, spleen, uterus | NA | NA |
| **Sc01.08.17** | M | A | Cerebrum, **CSF**, pharyngeal tonsils, lung, tracheobronchial lymph node, spleen, testis | Cerebrum, cerebellum, **spinal cord (33.96)**, pharyngeal tonsils, lung, tracheobronchial lymph node, liver | NA | NA |
| **Tt18.08.17** | F | A | Cerebrum, lung, tracheobronchial lymph node, spleen, kidney, uterus | Cerebrum, pharyngeal tonsils, lung, tracheobronchial lymph node, liver, spleen, uterus | NA | NA |
| **Sc26.04.18** | F | C | **Cerebrum,** lung, tracheobronchial lymph node, spleen | Cerebrum, **cerebellum (33,56)**, **spinal cord (35,85)**, pharyngeal tonsils, lung, tracheobronchial lymph node, liver, spleen | NA | NA |
| **Tt28.04.18** | M | A | Cerebrum, tracheobronchial lymph node, spleen, kidney, testis | Cerebellum, spinal cord, pharyngeal tonsils, tracheobronchial lymph node, liver, spleen, testis | NA | NA |
| **Sc26.07.19** | M | J | Cerebrum, lung, tracheobronchial lymph node, spleen, kidney, testis | **Cerebellum (35,20)**, spinal cord, tracheobronchial lymph node, lung, spleen, testis | NA | NA |
| **Sc01.08.19** | F | J | **Cerebrum,** lung, **tracheobronchial lymph node**, spleen, kidney, uterus | Cerebrum, pharyngeal tonsils, tracheobronchial lymph node, liver, uterus | NA | NA |
| **Sc01.03.21** | M | A | **Cerebrum**, **CSF**, lung, tracheobronchial lymph node, urinary bladder, uterus | **Cerebrum (34.46)**, **cerebellum (36.36)**, **CSF (31.47)**, **pharyngeal tonsils (36.97)**, **lung (36.86)**, tracheobronchial lymph node, **uterus (36.58)**, **liver (37.33)**, **spleen (36.03)** | NA | NA |
| **Sc04.05.21** | F | A | Cerebrum, **tracheobronchial lymph node**, kidney, urinary bladder, ovary, uterus | Cerebrum, cerebellum, **pharyngeal tonsils (33.34)**, **tracheobronchial lymph node (37.52)**, liver, spleen, uterus | NA | NA |
| **Sc06.08.21** | M | J | Cerebrum, **CSF**, lung, tracheobronchial lymph node, kidney, urinary bladder, testis | Cerebrum, **cerebellum (36.34)**, **CSF (32.95)**, **pharyngeal tonsils (39.43)**, lung, liver, spleen | NA | NA |
